# A Two-Step Annealing Method to Enhance the Pyroelectric Properties of Mn:PIMNT Chips for Infrared Detectors

**DOI:** 10.3390/ma13112562

**Published:** 2020-06-04

**Authors:** Rongfeng Zhu, Jing Zhao, Jianwei Chen, Bijun Fang, Haiqing Xu, Wenning Di, Jie Jiao, Xi’an Wang, Haosu Luo

**Affiliations:** 1 Key Laboratory of Inorganic Functional Materials and Devices, Shanghai Institute of Ceramics, Chinese Academy of Sciences, Shanghai 201800, China; zhurongfeng@student.sic.ac.cn (R.Z.); zhaojing@student.sic.ac.cn (J.Z.); xuhaiqing@mail.sic.ac.cn (H.X.); dwn@mail.sic.ac.cn (W.D.); jiejiao@mail.sic.ac.cn (J.J.); 2 Center of Materials Science and Optoelectronics Engineering, University of Chinese Academy of Sciences, Beijing 100049, China; 3 School of Materials Science and Engineering, Jiangsu Collaborative Innovation Center of Photovolatic Science and Engineering, Jiangsu Province Cultivation Base for State Key Laboratory of Photovoltaic Science and Technology, National Experimental Demonstration Center for Materials Science and Engineering, Changzhou University, Changzhou 213164, China; fangbj@cczu.edu.cn

**Keywords:** Mn:PIMNT crystals, two-step annealing, defects, surface stress, infrared detectors

## Abstract

Mn:0.15Pb(In_1/2_Nb_1/2_)O_3_-0.55Pb(Mg_1/3_Nb_2/3_)O_3_-0.30PbTiO_3_ (Mn:PIMNT) pyroelectric chips were prepared by a two-step annealing method. For the two steps, annealing temperatures dependence of microstructure, defects, surface stress, surface roughness, dielectric properties and pyroelectric properties were studied comprehensively. The controlling factors influencing the pyroelectric properties of the Mn:PIMNT crystals were analyzed and the optimum annealing temperature ranges for the two steps were determined: 600–700 °C for the first step and 500–600 °C for the second step. The pyroelectric properties of the thin Mn:PIMNT chips were significantly enhanced by the two-step annealing method via tuning oxygen vacancies and eliminating surface stress. Based on Mn:PIMNT pyroelectric chips annealed at the most favorable conditions (annealed at 600 °C for the first step and 500 °C for the second step), infrared detectors were prepared with specific detectivity D* = 1.63 × 10^9^ cmHz^1/2^W^−1^, nearly three times higher than in commercial LiTaO_3_ detectors.

## 1. Introduction

During the past few decades, pyroelectric infrared detectors have attracted extensive attention in the field of infrared detection technology due to their prominent features of high sensitivity, no requirement for cooling, wide-band frequency response, fast response speed, etc. [1,2,3,4,5,6]. Specific detectivity (D*), which is a form of signal-to-noise ratio, is one of the most important parameters that characterize the performance of pyroelectric infrared detectors [7,8,9]. Therefore, there are two ways to improve the D* of pyroelectric infrared detectors: one is to improve responsivity (*R_V_*), which is closely related to the pyroelectric coefficient (*p*) and heat capacity (*H_p_*) of the pyroelectric materials; the other is to reduce noise (*N*), which is dominated by different noise mechanisms under different frequencies [10,11,12].

According to the dominant noise at different frequency ranges, D* can be simplified, meaning that ignoring the influence of the back-end circuit, the D* of different frequency bands is mainly affected by three figures of merit (FOMs) of the pyroelectric materials, i.e., the FOMs for current responsivity *F_i_* ($F_{i}=\frac{p}{C_{V}}$), detectivity *F_d_* ($F_{d}=\frac{p}{C_{v}{\left(\varepsilon_{r}\tan\delta \right)}^{1/2}}$) and voltage responsivity *F_v_* ($F_{v}=\frac{p}{C_{V}\varepsilon_{r}}$) [13,14,15]. The thickness (*d*) and area (*A_s_*) of the pyroelectric chips also exert great influence on D* [16]. Currently, relaxor ferroelectric single crystals, such as Pb(Mg_1/3_Nb_2/3_)O_3_-PbTiO_3_ (PMNT) and Pb(In_1/2_Nb_1/2_)O_3_-Pb(Mg_1/3_Nb_2/3_)O_3_-PbTiO_3_ (PIMNT), are considered to be one of the most promising next-generation pyroelectric materials due to their much higher pyroelectric coefficient (*p*), lower dielectric loss (*tanδ*), adaptable dielectric constant (*ε_r_*) and relatively low specific heat (*C_V_*) compared to commercial pyroelectric materials, such as lithium tantalate (LaTiO_3_, LT) crystals and lead zirconate titanate (PbZr_x_Ti_(1−x)_O_3_, PZT) ceramics [17,18,19,20,21,22].

Extensive research has been carried out indicating the practical applications of relaxor ferroelectric single crystals in pyroelectric infrared detectors [23,24,25,26,27,28,29]. Y. Tang et al. revealed that PMNT single crystals along the [111] direction with a PbTiO_3_ (PT) content of 26% present the best pyroelectric performance, with *F_d_*= 15.3 × 10^−5^ Pa^−1/2^, via systematically studying the effects of composition, crystallographic orientation, electric field, frequency and aging on the pyroelectric properties of PMNT single crystals [24,25]. L. Liu et al. determined that the *tanδ* of PMNT(74/26) single crystals can be effectively suppressed by the doping of Mn^2+^ with *F_d_*= 40.2 × 10^−5^ Pa^−1/2^, which is the result of the pinning effect of the ${\left(Mn^{2+}\right)}_{Ti}^{\prime \prime }-V_{O}^{••}$ dipoles verified by X. Li et al. [26,27]. Due to the relatively low Curie temperature of PMNT crystals (*T_C_/T_m_*, almost 120 °C for Mn:PMNT(74/26) crystals), PIMNT crystals, a PMNT-based ternary system, with higher *T_C_/T_m_* have also been investigated by many researchers to improve operating temperature and ensure temperature stability [28,29].

The annealing process exerts great influence on the microstructure and electrical properties of materials [30,31,32]. Whether single crystals, ceramics or thin films, the annealing process has been shown to improve performance [30,31,32]. By studying the effect of different annealing atmospheres on pyroelectric properties, L. Li showed that oxygen annealing displays the best effect on improving the pyroelectric properties of PIMNT single crystals [33]. However, this research did not take the influence of the annealing temperature into account. In addition, not only the bulk materials but also the prepared thin pyroelectric chips need annealing, that is, the as-grown relaxor ferroelectric single crystals need annealing to tune oxygen vacancies and the prepared thin pyroelectric chips need annealing to eliminate surface stress after thinning and polishing [34]. Although the annealing process for thin pyroelectric chips can play a role in tuning oxygen vacancies, the annealing process for as-grown crystals is also indispensable because the required annealing conditions for tuning oxygen vacancies and eliminating stress are different. Additionally, besides enhancing pyroelectric properties, tuning oxygen vacancies can also improve the integrity of single crystals, which is necessary for the prepared thin pyroelectric chips to avoid breaking in the process of thinning and polishing.

In this work, a two-step annealing method was adopted to prepare pyroelectric chips based on PIMNT single crystals. To enhance the detectivity of the PIMNT chips, the effects of annealing temperature on pyroelectric properties and microstructure in two-step annealing were studied. The optimized annealing temperature ranges for two-step annealing were determined and the mechanisms were explained at the micro-level.

## 2. Materials and Methods

Modified Bridgman process-developed Mn:PIMNT single crystals (nominal composition: 0.15Pb(In_1/2_Nb_1/2_)O_3_-0.55Pb(Mg_1/3_Nb_2/3_)O_3_-0.30PbTiO_3_ doped by 1 mol% Mn) were grown and cut along the [111] direction. Crystal samples from the same plate (thickness of 0.5 mm) were annealed at 400, 500, 600, 700 and 800 °C for 10 h (O_2_—8 × 10^4^ Pa), respectively (first-step annealing). The thick unannealed Mn:PIMNT crystals can be described as as-grown crystals, since the crystals have not undergone any post-treatment process. X-ray diffractometry (XRD, Rigaku D/max-2500/PC X-ray Diffractometer, Rigaku Corp., Tokyo, Japan) was used to investigate the crystal structure of the as-grown and annealed Mn:PIMNT crystals using crystal powder [35,36,37]. After sputtering the electrodes, the electrical properties of the Mn:PIMNT crystals with dimensions of 4 mm × 4 mm were measured and calculated [29,30,31]. Parts of the Mn:PIMNT crystals after 600 °C annealing with no other treatment were then thinned and polished to 20 μm by silicon carbide powder (diameter of 3.5–7 μm) and acidic silica sol (diameter of 50 nm). The polished thin Mn:PIMNT crystals were also annealed at 400, 500, 600, 700 and 800 °C for 10 h (O_2_—8 × 10^4^ Pa), respectively (second-step annealing). An MFP-3D piezoresponse force microscope (PFM) was used to characterize the morphology and domain structures of the unannealed and annealed thin Mn:PIMNT pyroelectric chips. The pyroelectric and dielectric properties of the thin Mn:PIMNT pyroelectric chips were also measured and calculated.

## 3. Results and Discussion

**Figure 1:**
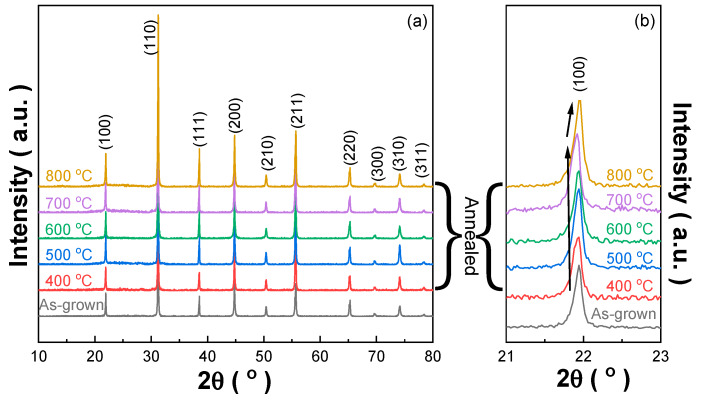
XRD patterns of the Mn:PIMNT crystal powder. (**a**) 10°–80°; (**b**) 21°–23° (expanded XRD patterns of the (100) diffraction peak). Abbreviations: Mn:PIMNT, Mn:0.15Pb(In_1/2_Nb_1/2_)O_3_-0.55Pb(Mg_1/3_Nb_2/3_)O_3_-0.30PbTiO_3_.

Figure 1 shows the X-ray powder diffraction (XRPD) patterns of the crystal powder from the as-grown and annealed Mn:PIMNT crystals, and the corresponding lattice parameters, calculated using the full diffraction profile fitting offered by the MDI Jade 6.5 software, are listed in Table 1. According to the ternary phase diagram, the Mn:PIMNT crystals we used (0.15Pb(In_1/2_Nb_1/2_)O_3_-0.55Pb(Mg_1/3_Nb_2/3_)O_3_-0.30PbTiO_3_) are located at the rhombohedral side around the morphotropic phase boundary (MPB), which can be further identified by the symmetric, single and sharp diffraction peaks [32]. From Table 1, it is obvious that the annealing treatment exerts great influence on the unit cell of Mn:PIMNT crystals, which gets larger after annealing. Furthermore, the unit cell exhibits a trend of constantly getting bigger with the raising of the annealing temperature (400–700 °C), but gets smaller at the highest annealing temperature (800 °C). Such change in the unit cell corresponds to the shift of the 2θ angle diffraction peaks based on the Bragg equation, which can be seen more clearly from the expanded (100) diffraction peak in Figure 1b, which may be attributed to variation in the defects in the Mn:PIMNT crystals. It is known that due to the low oxygen partial pressure at high temperatures (>1000 °C), there is generally a certain concentration of oxygen vacancies in the growth of oxide crystals, including Mn:PIMNT crystals [30,31,32]. After annealing in an oxygen-rich atmosphere, the oxygen vacancies in Mn:PIMNT crystals can be decreased, inducing the recovery of the distorting lattice, i.e., the augment of the unit cell of the Mn:PIMNT crystals. In addition, due to the thermal activation characteristics of the defects, the higher the annealing temperature, the more easily the oxygen defects will move and the more oxygen defects in the crystals will be compensated. However, as the annealing temperature reaches 800 °C, the lead ions (Pb^2+^) begin to volatilize, causing the unit cell of the Mn:PIMNT crystals to shrink.

**Figure 2:**
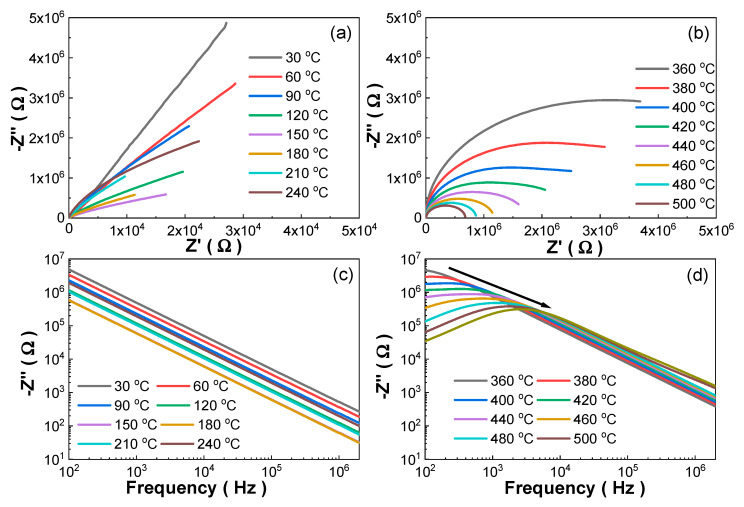
Complex impedance spectra of the as-grown Mn:PIMNT crystals measured from 100 Hz to 2 MHz at different temperatures: (**a**,**b**) imaginary parts (Z″) versus real parts (Z′) and (**c**,**d**) imaginary parts (Z″) versus frequency.

**Figure 3:**
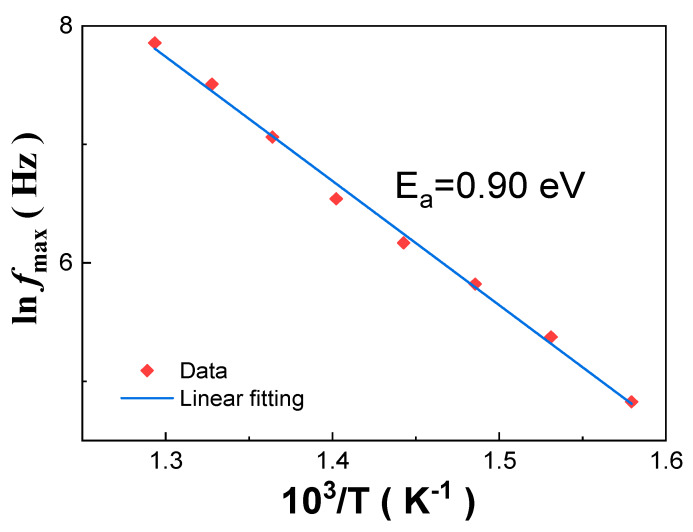
Linear fitting of relaxation frequency (*f_max_*) of the as-grown Mn:PIMNT single crystals according to the Arrhenius law.

Figure 2 presents the complex impedance spectra of the as-grown Mn:PIMNT crystals measured from 100 Hz to 2 MHz at different temperatures. Note that the impedance spectra at low temperatures (Figure 2a,c) are different from those at high temperatures (Figure 2b,d), which can be explained by the different conduction mechanisms and relaxation mechanisms at different temperatures. The different conduction and relaxation mechanisms are associated with different defects in materials, which are thermally activated at different temperatures and need different activation energy (*E_a_*) to move [36,37]. The activation energy (*E_a_*) of the movement of the dominant defects, i.e., the main controlling factor for the conduction and relaxation mechanisms can be calculated according to the Arrhenius law [38,39]. Therefore, it is an effective method for inferring the change in defects in the Mn:PIMNT crystals based on the calculated activation energy (*E_a_*). From Figure 2d, relaxation frequencies (*f_max_*) at different temperatures, i.e., frequencies corresponding to the top of −Z′′ at each temperature, can be obtained. According to the Arrhenius law, $f_{\max}=f_{0}\exp(-E_{a}/k_{B}T)$, relaxation frequency (*f_max_*) versus Kelvin temperature (*T*) can be plotted and linear-fitted (Figure 3) [36,38]. The activation energy of the as-grown Mn:PIMNT crystals at high temperatures is 0.90 eV, nearly 1 eV, which corresponds with the hopping of the oxygen vacancies $V_{O}^{••}$, indicating that the oxygen vacancies dominate the relaxation mechanisms. The activation energy of the Mn:PIMNT crystals annealed at different temperatures is also calculated and listed in Table 2. The corresponding complex impedance spectra and the detailed fitting procedure of other Mn:PIMNT crystals annealed at different temperatures are in Supplementary Materials (Figures S1–S5). All the coefficient of determination (*Adj. R-Square*) values of the as-grown Mn:PIMNT crystals characterizing the goodness of fit are higher than 0.99, close to 1, indicating the good fit of the Arrhenius law. Compared to the as-grown Mn:PIMNT crystals, the activation energy of the Mn:PIMNT crystals after annealing shows an obvious increase. Such increase can be attributed to variation in the dominant defects from the oxygen vacancies $V_{O}^{••}$ to the ${\left(Mn^{2+}\right)}_{Ti}^{\prime \prime }-V_{O}^{••}$ dipoles, due to the decline of the oxygen vacancies [36,38,39,40,41]. Note that the Mn:PIMNT crystals annealed at 600 and 700 °C exhibit the highest activation energy, both being 1.57 eV, indicating that higher temperature has little effect on compensating for the oxygen vacancies when the annealing temperature reaches 600 °C, which can also be identified by the close cell volumes calculated in Table 1 (66.09 Å^3^ for 600 °C and 66.10 Å^3^ for 700 °C). The slight decrease in activation energy for the Mn:PIMNT crystals annealed at 800 °C may result from the increase in lead vacancies due to the evaporation of lead ions, inducing easier hopping of the lead vacancies.

**Figure 4:**
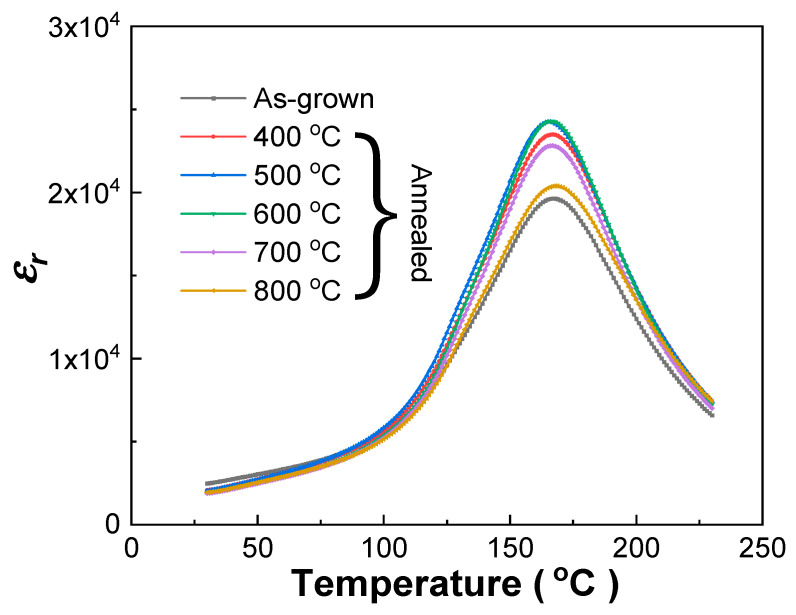
Dielectric constant (1 kHz) versus temperature of the unpoled Mn:PIMNT single crystals annealed at different temperatures upon heating.

The dielectric constants (1 kHz) of the unpoled as-grown and annealed Mn:PIMNT crystals measured at different temperatures are presented in Figure 4, from which some corresponding parameters were extracted and are listed in Table 3. In Figure 4 and Table 3, the Curie temperature (*T_C_/T_m_*) of the crystals annealed at different temperatures is basically unchanged, being nearly 167 °C, almost 47 °C higher than that of the binary Mn:PMNT(74/26) crystals. Dielectric properties are greatly affected by the annealing temperatures. As the annealing temperature increases, the dielectric constant *ε_r_* near room temperature (30 °C) firstly exhibits a constant downward trend and finally increases at 800 °C, while the dielectric constant *ε_m_* at the Curie temperature firstly continuously rises and finally declines at 800 °C, which is likely to also be influenced by the variation in oxygen vacancy and lead vacancy defects in the crystals.

**Figure 5:**
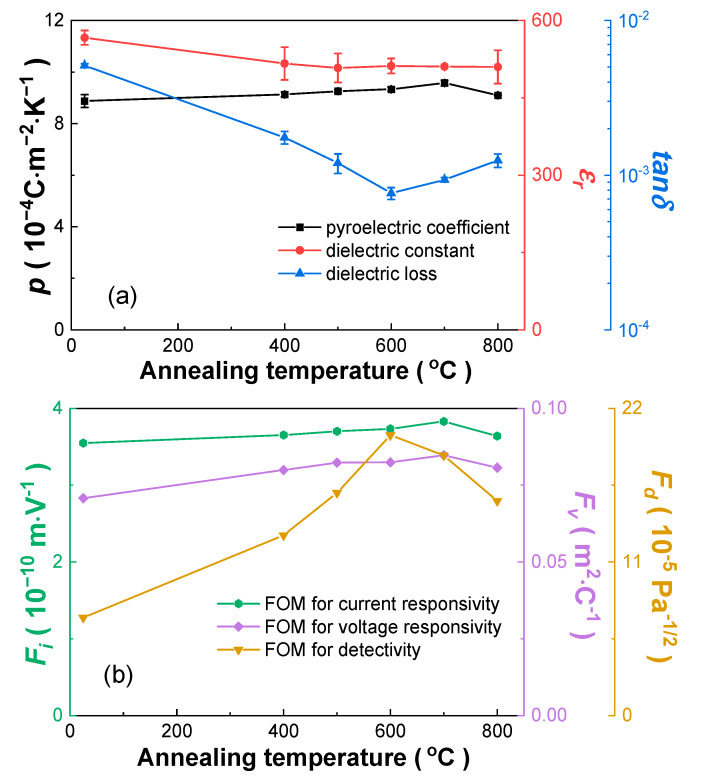
Room temperature-measured dielectric and pyroelectric properties versus annealing temperature of the thick Mn:PIMNT crystals. (**a**) *p*, *ε_r_* and *tanδ*; (**b**) *F_i_*, *F_v_* and *F_d_*.

Figure 5 displays the dielectric properties (*ε_r_* and *tanδ* measured at 1 kHz after poling) and pyroelectric properties (*p*, *F_i_*, *F_v_* and *F_d_*) of the thick Mn:PIMNT crystals annealed at different temperatures. With the increase in annealing temperature, *p* firstly continuously increases, and finally decreases at 800 °C, which is also consistent with the variation of the defects in the Mn:PIMNT crystals. Unlike the unpoled Mn:PIMNT crystals, *ε_r_* for the poled ones decreases after annealing, but the effect of the annealing temperature is not significant. Furthermore, the variation trend in *tanδ* is slightly different from in the defects. As the annealing temperature rises, *tanδ* constantly decreases at the beginning, but rises from 700 °C, not 800 °C. Such difference may be correlated with the weakening pinning effect of the ${\left(Mn^{2+}\right)}_{Ti}^{\prime \prime }-V_{O}^{••}$ dipoles, resulting from the further decline in oxygen vacancies when decreasing to a certain level [27]. Combined with the variation in the defects and the pyroelectric properties of the Mn:PIMNT crystals, the annealing temperature at the first step can be determined to be in a range of 600–700 °C. The thick Mn:PIMNT crystals annealed at 600 °C exhibit the highest *F_d_*, with *p* = 9.33 × 10^−4^ Cm^−2^K^−1^, *ε_r_* = 512, *tanδ* = 0.076%, *F_i_* = 3.73 mV^−1^, *F_v_*= 0.082 m^2^C^−1^ and *F_d_* = 20.1 Pa^−1/2^, while the ones annealed at 600 °C present the highest *p*, *F_i_* and *F_v_*, with *p* = 9.57 × 10^−4^ Cm^−2^K^−1^, *ε_r_* = 511, *tanδ* = 0.093%, *F_i_* = 3.83 mV^−1^, *F_v_* = 0.085 m^2^C^−1^ and *F_d_* = 18.6 Pa^−1/2^. In order to facilitate the following experiments, the Mn:PIMNT crystals were prepared after 600 °C annealing with no other treatment, which can effectively tune the oxygen vacancies to improve pyroelectric properties and improve the integrity of single crystals to avoid breaking during the thinning and polishing process.

**Figure 6:**
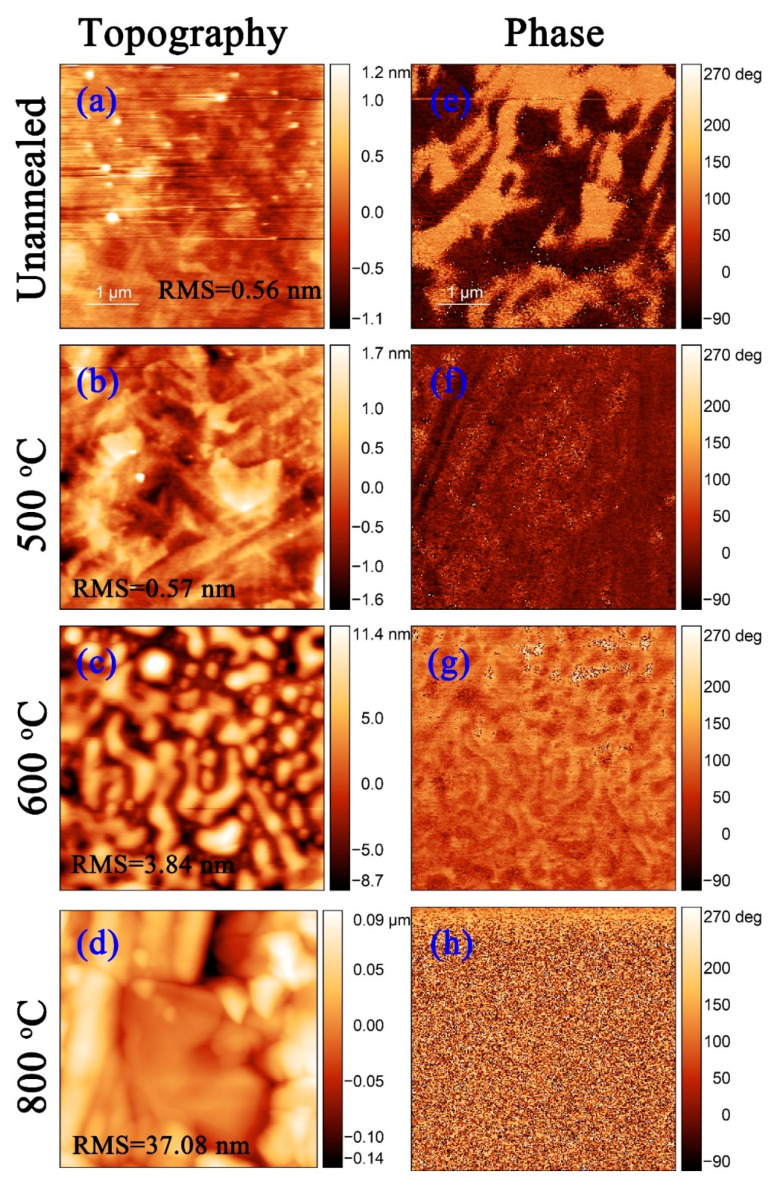
The PFM images of the thin polished Mn:PIMNT pyroelectric chips annealed at different temperatures: (**a–d**) topography (RMS value marked refers to the surface roughness root mean square (RMS)); (**e–h**) phase. Abbreviations: PFM, piezoresponse force microscope.

The PFM images of the thin polished Mn:PIMNT pyroelectric chips annealed at different temperatures are given in Figure 6. From the topography PFM images, it can be found that from unannealed to 800 °C annealing, the surface roughness root mean square (RMS) of the Mn:PIMNT pyroelectric chips increases (Figure 6a–d), indicating the thermal etching feature of annealing. Additionally, from the phase PFM images, as the annealing temperatures increase, the domain structures constantly change (Figure 6e–h), from island domains (Figure 6e) to strip domains (Figure 6f), then to mosaic domains (Figure 6g) and finally to invisible (Figure 6h). Such variation in the domain structures can be attributed to the different thermal etching rates of the domains.

**Figure 7:**
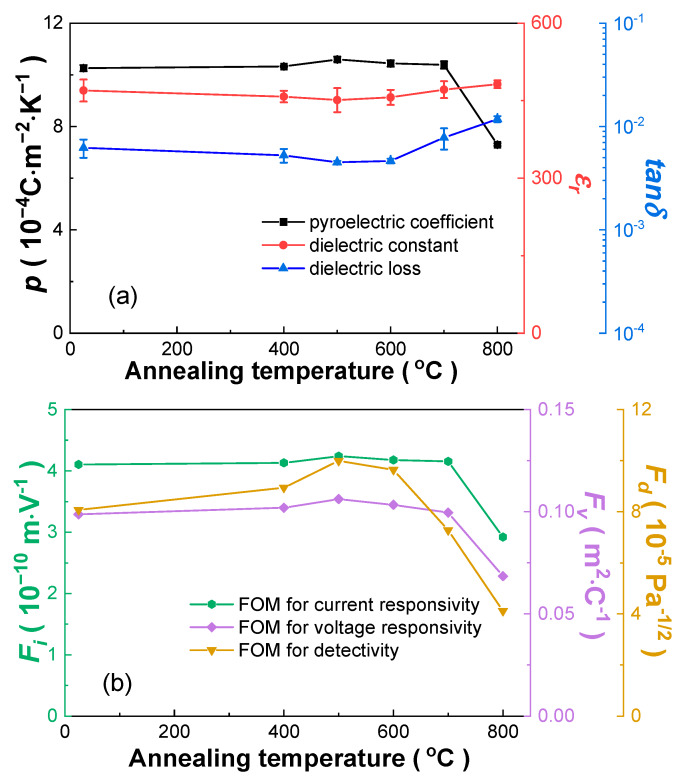
Room temperature-measured pyroelectric and dielectric properties versus annealing temperature of the thin Mn:PIMNT pyroelectric chips. (**a**) *p*, *ε_r_* and *tanδ*; (**b**) *F_i_*, *F_v_* and *F_d_*.

Figure 7 displays the dielectric properties (*ε_r_* and *tanδ* measured at 1 kHz after poling) and pyroelectric properties (*p*, *F_i_*, *F_v_* and *F_d_*) of the thin Mn:PIMNT pyroelectric chips annealed at different temperatures. Note that the electrical properties of the thin Mn:PIMNT pyroelectric chips are slightly different from those of the thick Mn:PIMNT crystals, which is related to the size effect and the surface effect [42,43,44,45,46,47]. The slightly higher pyroelectric coefficient of the thin Mn:PIMNT pyroelectric chips can be attributed to the measurement characteristic of the dynamic method, which measures the temperature change of the samples by measuring that of the sample stage. For the thick Mn:PIMNT crystals, the actual temperature change is lower than the measured temperature change due to the thickness, meaning that the obtained pyroelectric coefficient is relatively lower than the actual value. The slightly lower *ε_r_* and higher *tanδ* are the result of the interaction of the surface damage layer and surface stress, which can be ignored in thick Mn:PIMNT crystals [43,45]. From Figure 7, with the increase in the annealing temperature, the pyroelectric properties (*p*, *F_i_*, *F_v_* and *F_d_*) of the Mn:PIMNT pyroelectric chips firstly improve, then decline, and the 500 °C annealing samples exhibit the best pyroelectric properties, with *p* = 10.60 × 10^−4^ Cm^−2^K^−1^, *ε_r_* = 451, *tanδ* = 0.233%, *F_i_* = 4.23 mV^−1^, *F_v_* = 0.106 m^2^C^−1^ and *F_d_* = 9.99 Pa^−1/2^. Such change can be attributed to the elimination of surface stress and increased roughness (Figure 6). For the thin Mn:PIMNT chips, the surface stress generated by the thinning and polishing process will pull the [111]-oriented domains perpendicular to the surface to the horizontal direction of the surface, resulting in the diminution of pyroelectric performance, which can be eliminated by the annealing process [43]. However, the increased roughness induced by the thermal etching feature of the annealing process will cause the pyroelectric performance of the thin Mn:PIMNT chips to deteriorate [34,43]. It can be seen that the pyroelectric properties of the Mn:PIMNT pyroelectric chips annealed at 800 °C decline suddenly due to the sharply increased surface roughness (RMS = 37.08 nm, Figure 6d). In view of possible errors in the experimental tests, the annealing temperature of the PIMNT pyroelectric chips can be determined to be in a range of 500–600 °C.

**Figure 8:**
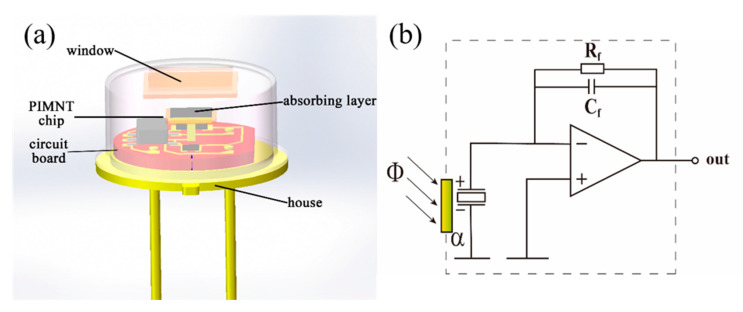
(**a**) Basic structure and (**b**) equivalent circuit (current mode) of the Mn:PIMNT pyroelectric infrared detectors.

Based on the Mn:PIMNT pyroelectric chips annealed at 500 °C, the pyroelectric infrared detectors were prepared. The basic structure and equivalent circuit of the detectors are displayed in Figure 8. The specific detectivity (D*) of the Mn:PIMNT infrared detectors is listed in Table 4, being 1.63 × 10^9^ cm·Hz^1/2^·W^−1^, nearly three times higher than in commercial LiTaO_3_ detectors. Although the detection ability is not as good as in Mn:PMNT detectors, the Mn:PIMNT detectors exhibit a higher Curie temperature *T_C_/T_m_* (higher than 40 °C), ensuring that they can be used stably at relatively higher temperatures.

## 4. Conclusions

A two-step annealing method was adopted to prepare the Mn:PIMNT pyroelectric chips. For first-step annealing, from as-grown to 800 °C annealing, the pyroelectric properties of thick Mn:PIMNT crystals are firstly continuously enhanced because of the constant decline in oxygen vacancies $V_{O}^{••}$, and finally deteriorate at 800 °C due to the volatilization of Pb^2+^, which can be verified by the variation in the unit cell and activation energy. The optimal temperature range for first-step annealing is 600–700 °C. For second-step annealing, from unannealed to 800 °C annealing, the pyroelectric properties of Mn:PIMNT pyroelectric chips firstly improve as a result of the elimination of surface stress, and then reduce due to the increase in surface roughness, which can be identified by PFM images. The ideal temperature range for second-step annealing is 500–600 °C. The specific detectivity (D*) of Mn:PIMNT-based pyroelectric infrared detectors (annealed at 600 °C for the first step and 500 °C for the second step) reaches 1.63 × 10^9^ cm·Hz^1/2^·W^−1^, nearly three times higher than in commercial LiTaO_3_ detectors.

## Figures and Tables

**Table 1:** Lattice parameters of the Mn:PIMNT crystals.

Annealing Conditions		a = b = c (Å)	α = β = γ (°)	Cell Volume (Å^3^)
**As-grown**		4.0376 (28)	89.903 (71)	65.82
**Annealed**	400 °C	4.0396 (26)	89.946 (67)	65.92
	500 °C	4.0415 (16)	90.004 (41)	66.01
	600 °C	4.0430 (17)	90.045 (43)	66.09
	700 °C	4.0433 (18)	90.062 (45)	66.10
	800 °C	4.0418 (04)	90.050 (12)	66.03

**Table 2:** The coefficient of determination (*Adj. R-Square*) for the fittings and activation energy (*E_a_*) for the relaxation processes of the as-grown and annealed Mn:PIMNT single crystals.

	As-Grown	Annealed				
		400 °C	500 °C	600 °C	700 °C	800 °C
***Adj. R-Square***	0.99517	0.99971	0.99985	0.99986	0.99977	0.99989
*E_a_* (eV)	0.90	1.45	1.53	1.57	1.57	1.55

**Table 3:** Dielectric parameters of the Mn:PIMNT crystals annealed at different temperatures.

	As-Grown	Annealed				
		400 °C	500 °C	600 °C	700 °C	800 °C
*ε_r_* (30 °C)	2.49 × 10^3^	2.08 × 10^3^	2.05 × 10^3^	1.90 × 10^3^	1.87 × 10^3^	1.94 × 10^3^
*T_C_/T_m_* (°C)	167	167	165	166	167	168
*ε_m_*	1.96 × 10^5^	2.35 × 10^5^	2.42 × 10^5^	2.43 × 10^5^	2.28 × 10^5^	2.03 × 10^5^

**Table 4:** Comparison of specific detectivity (D*) of the Mn:PIMNT pyroelectric infrared detectors and some other pyroelectric detectors (modulation frequency of 10 Hz, measured distance of 10 cm, measured bandwidth of 1 Hz, room temperature).

Mode	Materials	d (μm)	*T_C_/T_m_* (°C)	D* (cm·Hz^1/2^·W^−1^)	Reference
Current mode	LiTaO_3_	27	620	5.40 × 10^8^	[42,48]
	Mn:PMNT(72/28)	20	124	2.21 × 10^9^	[42,43]
	Mn: PIMNT(15/55/30)	20	167	1.63 × 10^9^	This work

## Supplementary Materials

The following are available online at https://www.mdpi.com/1996-1944/13/11/2562/s1, Figure S1: (a) Imaginary parts (Z″) versus real parts (Z′) measured from 100 Hz to 2 MHz, (b) imaginary parts (Z″) versus frequency measured from 100 Hz to 2 MHz and (c) linear fitting of relaxation frequency (*f_max_*) of the Mn:PIMNT single crystals annealed at 400 °C, Figure S2: (a) Imaginary parts (Z″) versus real parts (Z′) measured from 100 Hz to 2 MHz, (b) imaginary parts (Z″) versus frequency measured from 100 Hz to 2 MHz and (c) linear fitting of relaxation frequency (*f_max_*) of the Mn:PIMNT single crystals annealed at 500 °C, Figure S3: (a) Imaginary parts (Z″) versus real parts (Z′) measured from 100 Hz to 2 MHz, (b) imaginary parts (Z″) versus frequency measured from 100 Hz to 2 MHz and (c) linear fitting of relaxation frequency (*f_max_*) of the Mn:PIMNT single crystals annealed at 600 °C , Figure S4: (a) Imaginary parts (Z″) versus real parts (Z′) measured from 100 Hz to 2 MHz, (b) imaginary parts (Z″) versus frequency measured from 100 Hz to 2 MHz and (c) linear fitting of relaxation frequency (*f_max_*) of the Mn:PIMNT single crystals annealed at 700 °C, Figure S5: (a) Imaginary parts (Z″) versus real parts (Z′) measured from 100 Hz to 2 MHz, (b) imaginary parts (Z″) versus frequency measured from 100 Hz to 2 MHz and (c) linear fitting of relaxation frequency (*f_max_*) of the Mn:PIMNT single crystals annealed at 800 °C.
