# Supplementary material for: A Two-Step Annealing Method to Enhance the Pyroelectric Properties of Mn:PIMNT Chips for Infrared Detectors
Source: Materials (Basel). 2020 Jun 4;13(11):2562. doi: 10.3390/ma13112562 (PMC7321455; doi:10.3390/ma13112562)
Supplement: Supplementary file 1 [file materials-13-02562-s001.pdf]

materials

Supplementary Materials: A Two-Step Annealing Method to Enhance the Pyroelectric Properties of the Mn:PIMNT Chips for Infrared Detectors

Rongfeng Zhu [1,2], Jing Zhao [1,2], Jianwei Chen [1,*], Bijun Fang [3], Haiqing Xu [1], Wenning Di [1], Jie Jiao[1], Xi'an Wang [1,*] and Haosu Luo [1,2,*]

[1] Key Laboratory of Inorganic Functional Materials and Devices, Shanghai Institute of Ceramics, Chinese Academy of Sciences, Shanghai 201800, China; zhurongfeng@student.sic.ac.cn (R.Z.), zhaojing@student.sic.ac.cn (J.Z.), xuhaiqing@mail.sic.ac.cn (H.X.), dwn@mail.sic.ac.cn (W.D.), jiejiao@mail.sic.ac.cn (J.J.)

[2] Center of Materials Science and Optoelectronics Engineering, University of Chinese Academy of Sciences, Beijing, 100049, China

[3] School of Materials Science and Engineering, Jiangsu Collaborative Innovation Center of Photovolatic Science and Engineering, Jiangsu Province Cultivation Base for State Key Laboratory of Photovoltaic Science and Technology, National Experimental Demonstration Center for Materials Science and Engineering, Changzhou University, Changzhou 213164, China; fangbj@cczu.edu.cn

* Correspondence: chenjw@mail.sic.ac.cn (J.C.), wang1972wang@hotmail.com (X.W.), hsluo@mail.sic.ac.cn (H.L.); Tel.: +86-21-59927572 (H.L.)

Received: 11 May 2020; Accepted: 2 June 2020; Published: 4 June 2020

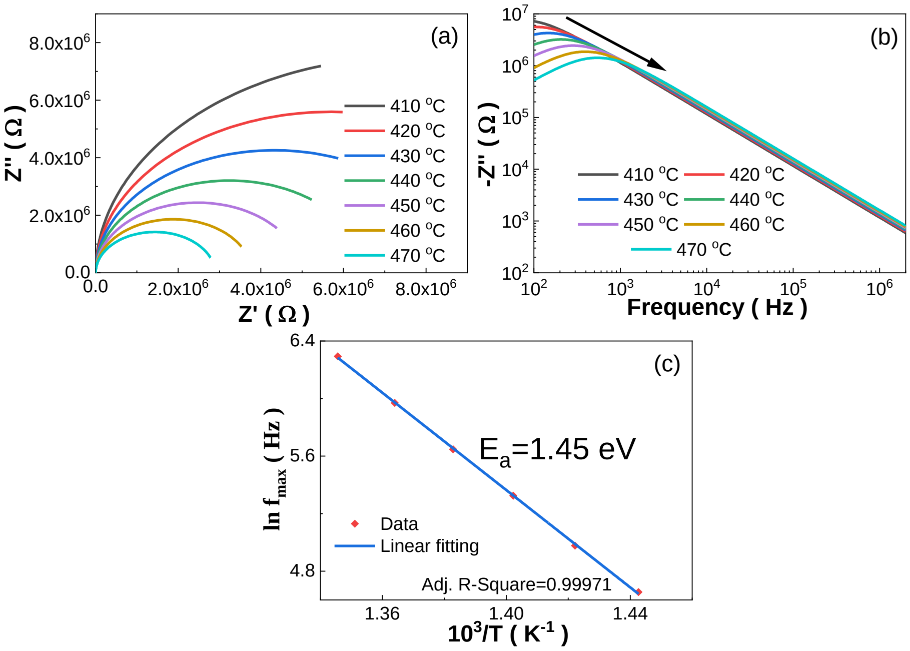

Figure S1. (**a**) Imaginary parts (Z″) versus real parts (Z′) measured from 100 Hz to 2 MHz, (**b**) imaginary parts (Z″) versus frequency measured from 100 Hz to 2 MHz and (**c**) linear fitting of relaxation frequency (f_{max}) of the Mn:PIMNT single crystals annealed at 400 °C.

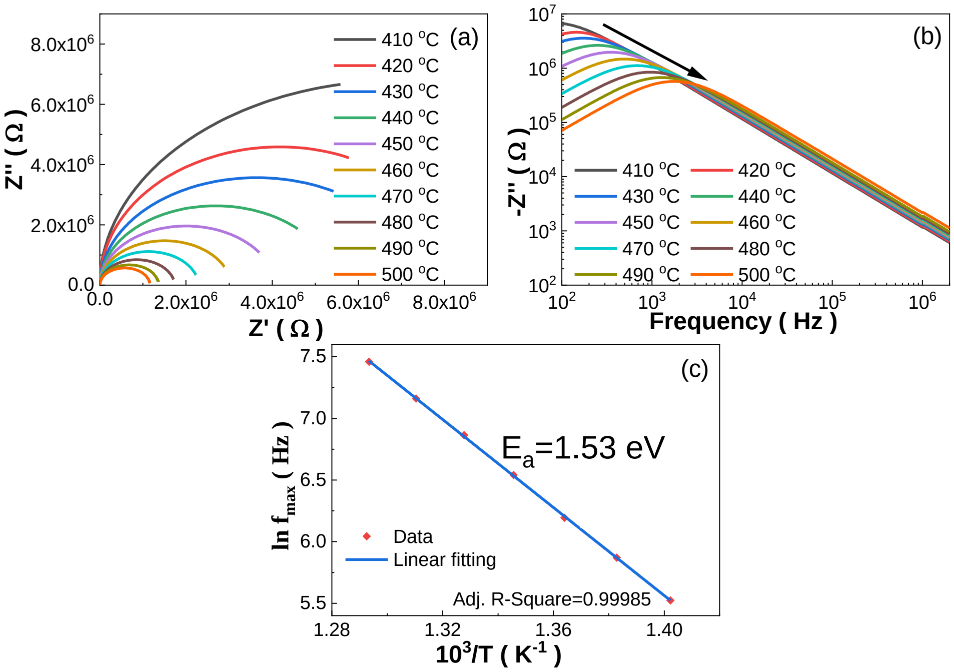

Figure S2. (**a**) Imaginary parts (Z″) versus real parts (Z′) measured from 100 Hz to 2 MHz, (**b**) imaginary parts (Z″) versus frequency measured from 100 Hz to 2 MHz and (**c**) linear fitting of relaxation frequency (f_{max}) of the Mn:PIMNT single crystals annealed at 500 °C.

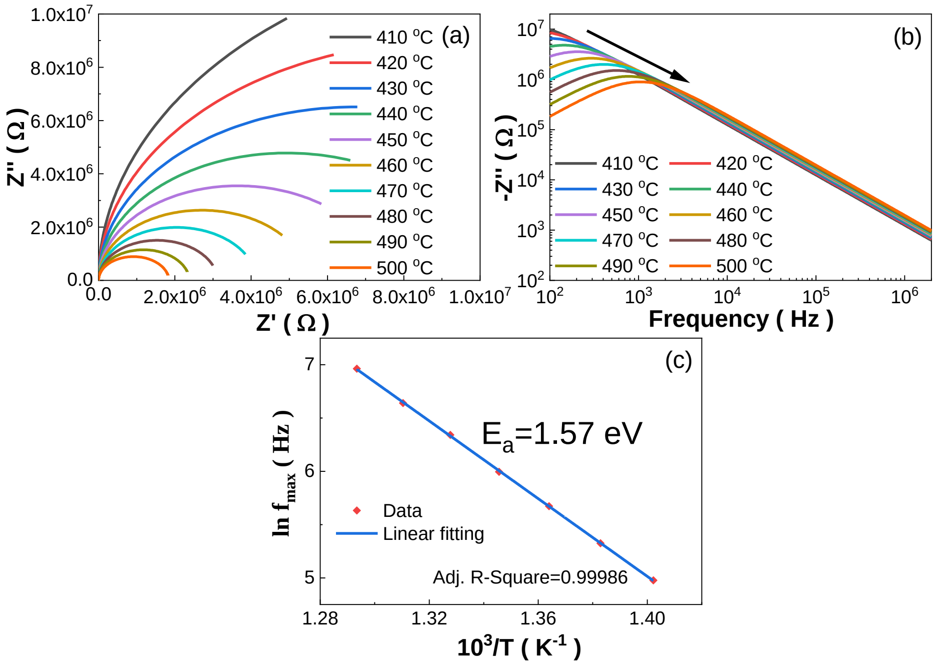

Figure S3. (**a**) Imaginary parts (Z″) versus real parts (Z′) measured from 100 Hz to 2 MHz, (**b**) imaginary parts (Z″) versus frequency measured from 100 Hz to 2 MHz and (**c**) linear fitting of relaxation frequency (f_{max}) of the Mn:PIMNT single crystals annealed at 600 °C.

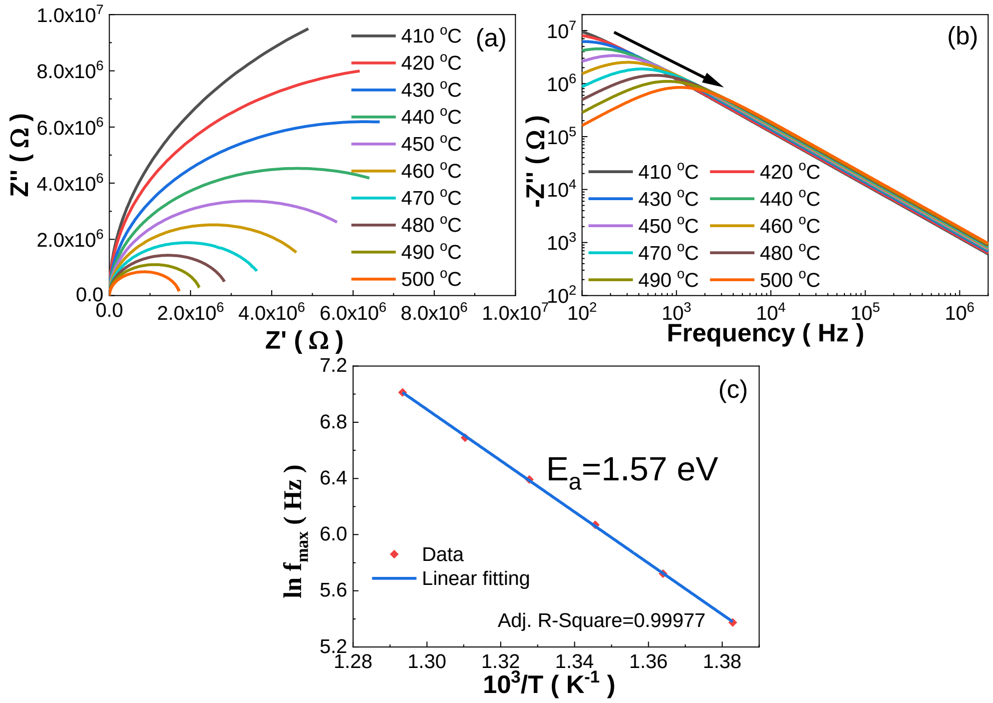

Figure S4. (**a**) Imaginary parts (Z″) versus real parts (Z′) measured from 100 Hz to 2 MHz, (**b**) imaginary parts (Z″) versus frequency measured from 100 Hz to 2 MHz and (**c**) linear fitting of relaxation frequency (f_{max}) of the Mn:PIMNT single crystals annealed at 700 °C.

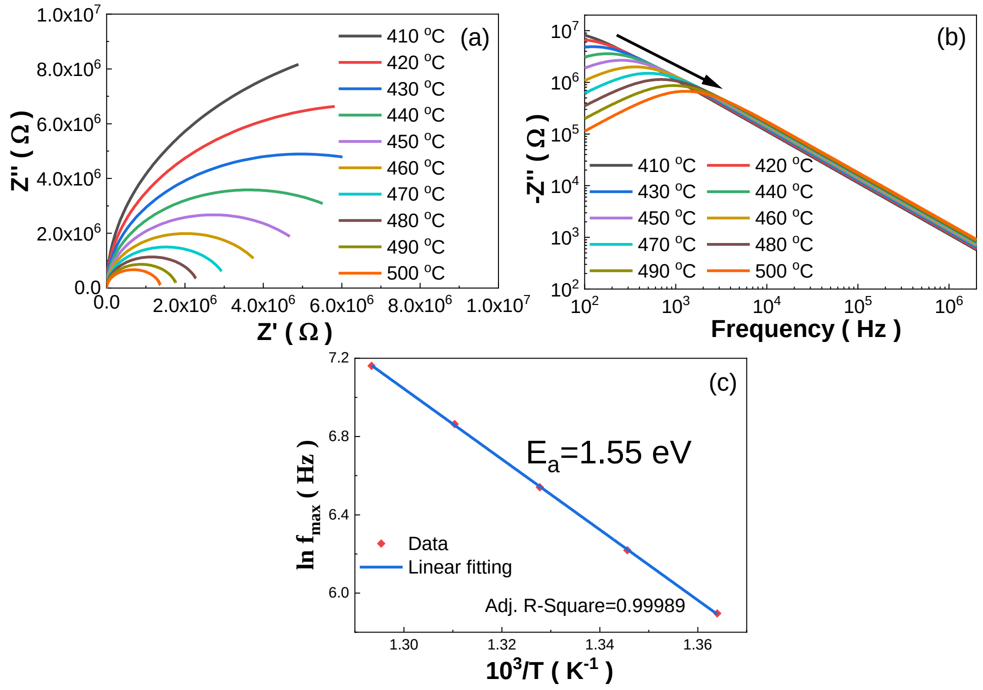

Figure S5. (**a**) Imaginary parts (Z″) versus real parts (Z′) measured from 100 Hz to 2 MHz, (**b**) imaginary parts (Z″) versus frequency measured from 100 Hz to 2 MHz and (**c**) linear fitting of relaxation frequency (f_{max}) of the Mn:PIMNT single crystals annealed at 800 °C.

© 2020 by the authors. Submitted for possible open access publication under the terms and conditions of the Creative Commons Attribution (CC BY) license (http://creativecommons.org/licenses/by/4.0/).
